# Correction to “Ex vivo drug responses and molecular profiles of 597 pediatric acute lymphoblastic leukemia patients”

**DOI:** 10.1002/hem3.70221

**Published:** 2025-09-18

Enblad AP, Krali O, Gezelius H, et al. Ex vivo drug responses and molecular profiles of 597 pediatric acute lymphoblastic leukemia patients. *HemaSphere*. 2025;9(7):e70176. doi:10.1002/hem3.70176

The supplemental methods mentioned in the subsection titled “DNA methylation and RNA‐sequencing data” was not included in the Supporting Information. This file has been added and cited at the applicable instance in the article.

We apologize for this error.

